# Genital allergy and irritant reactions in atopics and non-atopics

**DOI:** 10.1186/2045-7022-5-S1-O14

**Published:** 2015-03-11

**Authors:** Roman Nowicki

**Affiliations:** 1 Dept of Dermatology, Venereology and Allergology Medical University of Gdansk, Gdansk, Germany

## 

Genital allergy or irritant reaction should be considered as a possible diagnosis in all patients with genital soreness or irritation for which no infection or dermatosis can be identified and in whom clinical manifestations remain unchanged or worsen with treatment. The term "contact dermatitis" denotes an acute or chronic inflammatory skin disorder caused by chemical/ physical irritants - irritant contact dermatitis (ICD) or allergens - allergic contact dermatitis (ACD). Despite their different mechanisms, they can be difficult to distinguish from one another at the clinical, histology, and even molecular levels. Clinical manifestations may be associated with irritants. They have the potential to disrupt membranes or interfere with metabolic processes in the epidermis or dermis. Chemical ICD (CICD) is either acute or chronic, which is usually associated with strong and weak irritants, respectively. Highly alkaline soaps, detergents, and cleaning products all have the common efffect of directly affecting the barrier properties of the epidermis. These effects include removing fat emulsion, inflicting cellular damage on the epithelium, and increasing the transepidermal water loss (TEWL) by damaging the horny layer water-binding mechanisms and damaging the DNA, which causes the layer to thin. The cosmetic products responsible for irritation are mainly liquid foam hygiene products. used too frequently and/or too long and/or badly rinsed. In woman, depilatory products can also be to blame. Some patients complain of irritation due to the perfume of scented toilet papers. Physical ICD (PICD) is a less researched form of ICD due to its various mechanisms of action and a lack of tests for its diagnosis. A complete patient history combined with negative allergic patch testing is usually necessary to reach a correct diagnosis. The simplest form of PICD results from prolonged rubbing, although the diversity of implicated irritants is far wider. Examples include paper friction and scratchy clothing. The anogenital region is often the site of ICD: in men, promoted by sweat and rubbing and in woman, by aggressive hygiene. ICD affects very young and very old patients more severely. The greatest single risk for ICD is a history of atopic dermatitis.

